# The Future of Dentistry

**Published:** 1877-09

**Authors:** W. H. Morgan


### THE FUTURE OF DENTISTRY.

BY W. H. MORGAN, D. D. S.

*Read before the Tennessee Dental Society, June 28th, 1877.*

MR. PRESIDENT, GENTLEMEN OF THE ASSOCIATION:—

The subject now deserving most attention, and on which depends more entirely the future of the Dental Profession, both as to its standing, and usefulness is dental education. The laxity at least in feeling upon this subject, is most manifest in this State, in the fact that so few, who are aspiring to enter its ranks, are availing themselves of the facilities afforded by our Dental Colleges, (facilities no where else to be found) to prepare themselves for their life work. So far as I have been able to learn, not more than three or four of these in our State were in attendance upon our Dental School the past winter.

Another evidence of this laxity is the known fact that it is a very common practice for men calling themselves dentists, some of whom have pledged themselves to a very different course, by signing our constitution, to which I refer you, to take students so called into their offices, and after a few months sometimes weeks, and at most a year or so, dub then *Doctor*, turn them out to prey upon the unsuspecting and credulous. And then we, (I mean the dental profession) soon fall into line, and cordially accord to them the assumed title of doctor. I say assumed, because if the title means anything, it means, that he who bears it has received a degree. This practice, and it is a fraud, has become so common, that we scarcely note it. What, I ask, would be thought of a preacher, who would assume the title of doctor upon whom the degree of D. D. had never been conferred? or the man who would assume to be batchelor of law, or arts, or master of arts, who had not received the degree in either case?

you would brand such a one as a fraud. I do not mean to say that all who thus assume a title, propose to commit a fraud, for the habit has become far too common for that, but technically it is a fraud, a false assumption. Do not understand me as assuming that none of this class are qualified to practice dentistry, as I do not so believe; but I do assume and confidently assert, that if all such practitioners had availed themselves of the advantages of a regular course in a dental school, in addition to the course of study taken, they would possess higher, very much higher qualifications in their profession than they do.

Another wrong, and not an insignificant one is, that this man is *doctored* by courtesy, and thus get the benefit of an unearned title, a title that cost some of his fellow-practitioners years of study and self-denial to obtain, while he has it by assuming it, by taking that which does not belong to him. Lest I be misunderstood I would have you make the proper distinction between qualifications and degrees, no attainments in any profession or calling can constitute a man a doctor, it does not make him an M. D. a D. D. S. a or D. D., or any other kind of a doctor, for the latter signifies a degree conferred by some person or body of men claiming to be legally authorized to do so. John Hunter was not a doctor, neither was Benjamin Brodie, Thomas Bell, Lafoulon D'Saribode, and many others who are gone in the past, but who are rendered immortal by their learning and achievements, the same may be said of Blake, and Nasmyth, and Fitch, and Tomes of our own times; these men, known and honored as far as civilization has reached, were not, or are not doctors, and would scorn to assume to be what they are not.

Some of our Associations have already taken action upon this subject, instructing their members to give the title to all whom it belongs, and to call no man doctor who has not received the degree; this is just, and being right, wrongs no man. I trust the day is near at hand, when all our associations will take like action.

Another evil of our times is the practice of many who can hardly be said to have an office or place of business or local habitation, but travel around through the country, peddling out their services very much as a peddler does his wares, soliciting purchases thereof, chaffering and bargaining off the same with very little reference to uniformity of fees. Against such a practice our code of ethics is most positive, this association having adopted the code of the National Association which says: "It is unprofessional * * * to go from house to house to solicit or perform operations."

Let us in the future require of all our students a thorough course of study, the attendance upon lectures in some respectable dental school, the promise at least of a strict compliance with our code of ethics, a strict compliance with the law as laid down in our constitution, I mean the constitution of this association; let us discourage the violation of every principle held in our code, and banded together thus uphold the dignity and honor of our calling; if we will do so, we may reasonably hope to see it advance, and in advancing command more fully the respect of our fellowmen; we may reasonably hope in the years to come to see it arise from its present demoralized condition, asserting its dignity, take its place beside the other learned professions, proving to the world the truth of what every dental association, so far as I know in the United States, has so formally and solemnly set forth in their adopted code, that dentistry is a specialty of medicine, and by implication is entitled to all the honors of such relationship.

